# Naringin Alleviates Autistic‐Like Behaviors in BTBR Mice Through Cannabinoid Receptor Type 1‐Mediated Restoration of Hippocampal Neurogenesis

**DOI:** 10.1111/cns.70654

**Published:** 2025-11-28

**Authors:** Yulong Liu, Meiling Xia, Xinggao Zhang, Jing Luo, Tianyao Liu, Hong Gong, Jiayin Liu, Mei Chen, Lian Wang, Jinghui Zhao, Meifeng Gong, Yi Luo, Xiaotang Fan

**Affiliations:** ^1^ Department of Military Cognitive Psychology, School of Psychology Third Military Medical University (Army Medical University) Chongqing China; ^2^ Department of Histology and Embryology Third Military Medical University Chongqing China; ^3^ Key Laboratory of Extreme Environmental Medicine Ministry of Education Chongqing China

**Keywords:** ASD, BTBR, CB1 receptor, hippocampus, naringin, neurogenesis

## Abstract

**Background:**

Naringin, a flavanone glycoside (naringenin 7‐O‐neohesperidose), exhibits a broad range of pharmacological activities, including neuroprotection. However, its effects on autistic‐like behavior have not been extensively studied.

**Methods:**

In this investigation, we utilized the autistic BTBR T + tf/J (BTBR) mice to conduct behavioral tests assessing autistic‐like phenotypes. We evaluated hippocampal neurogenesis through immunofluorescence and employed molecular biological techniques, along with RNA sequencing, to elucidate the underlying molecular mechanisms.

**Results:**

Our findings revealed that the administration of naringin alleviated autism‐associated behaviors in BTBR mice. RNA sequencing analysis indicated that naringin facilitated the recovery of impaired hippocampal neurogenesis in these mice, as evidenced by an increase in doublecortin (DCX)‐positive cells and neuronal progenitor cells (NPCs) in the dentate gyrus (DG). Furthermore, we confirmed that the cannabinoid receptor type‐1 (CB1) plays a role in the therapeutic effects of naringin.

**Conclusions:**

This research highlights the potential of naringin as a promising treatment option for autism spectrum disorder (ASD) and suggests that targeting hippocampal neurogenesis through the CB1 receptor may be an effective strategy.

## Graphical abstract

Naringin treatment can alleviate core symptoms associated with ASD, leading to improved sociability, correction of social cognition deficits, and reduced repetitive stereotypical behaviors. In terms of specific mechanisms, naringin promotes hippocampal neurogenesis by inhibiting retrograde endocannabinoid signaling, which is achieved by reducing CB1 receptor expression.

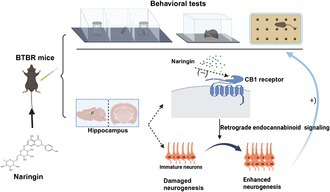

**Abbreviations**
ACEA: arachidonyl‐2′‐chloroacetamide
ASD: autism spectrum disorder
CB1: cannabinoid receptor type‐1
CC: cellular component
DCX: doublecortin
DEGs: differentially expressed genes
DG: dentate gyrus
EACAMU: Experimental Animal Centre of the Army Medical University
eCBs: endocannabinoid signaling
GCL: granule cell layer
GFAP: glial fibrillary acidic protein
GO: Gene Ontology
GPCR: G protein‐coupled receptor
KEGG: Kyoto Encyclopedia of Genes and Genomes
LSD: least significant difference
LXR: Liver X Receptor
MF: Molecular Function
NAR: naringin
NOR: Novel Object Recognition
NPCs: neural progenitor cells
RGCs: radial glial cells
SAL: saline
SGZ: subgranular zone
SOX2: SRY‐box transcription factor 2
VPA: valproic acid

## 1 Introduction

Autism spectrum disorder (ASD) encompasses a diverse range of pervasive neurodevelopmental disorders characterized by deficits in social interaction, restricted interests, and stereotyped behaviors [1]. The etiology of ASD involves the interplay between genetic and environmental factors [2], though the pathogenesis and underlying molecular mechanisms remain largely unclear [3]. The rising prevalence of autism presents significant medical and social challenges that necessitate immediate attention. Currently, behavioral interventions and antipsychotic medications constitute the primary treatment modalities for individuals with ASD [4]. However, there are no pharmacological treatments that effectively address the core symptoms of the disorder. Consequently, there is an urgent need for further investigation to identify more effective interventions and to advance the development of personalized treatment strategies for children with ASD.

The subgranular zone (SGZ) of the dentate gyrus (DG) is populated with neural progenitor cells (NPCs) that continuously generate new neurons throughout an individual's lifespan [5]. This ongoing neurogenesis in the hippocampus necessitates significant remodeling of existing neural circuitry, which plays a crucial role in cognitive processes and mood regulation [6]. Recent research has highlighted the significance of hippocampal neurogenesis, particularly within the DG, in the pathophysiology of ASD, as impaired neurogenesis has been linked to cognitive and behavioral deficits observed in the disorder [7]. Importantly, boosting hippocampal neurogenesis could be a target for treating ASD, as evidenced by our previous studies [8, 9].

Naringin is predominantly found in grapefruit and various other citrus species [10]. It is known for its extensive range of biological activities, including antioxidant properties, antifibrotic effects, anti‐inflammatory actions, metabolic modulation, and the activation of cholinergic transmission [11, 12]. Studies have demonstrated that naringin can protect against different types of brain damage in various animal models, encompassing subarachnoid hemorrhage [13], traumatic brain injury [14], cerebral ischemia–reperfusion injury [15], and spinal cord injury [16]. Notably, naringin has been shown to reverse the suppression of neurogenesis in the hippocampus's DG in hyperglycemic mice [17]. Furthermore, naringin exhibits antidepressant and anxiolytic effects by promoting hippocampal neurogenesis through the activation of CREB signaling [18]. Given naringin's role in promoting neurogenesis, we hypothesize that its ability to alleviate behavioral deficits may be linked to enhancing hippocampal neurogenesis in models of ASD. It is worth investigating whether naringin could improve autistic behaviors by regulating neurogenesis in the hippocampus.

This study demonstrates that treatment with naringin significantly mitigates ASD‐like behaviors in BTBR mice. Our findings also showed that naringin administration restores impaired hippocampal neurogenesis, as evidenced by an increase in doublecortin (DCX)‐positive cells and NPCs in the DG. Furthermore, we identified the cannabinoid receptor 1 (CB1) as a key mediator of naringin's therapeutic effects, suggesting a potential role for the endocannabinoid system involved in its therapeutic action. This study highlights the potential of naringin as a novel therapeutic agent for ASD and underscores the importance of targeting hippocampal neurogenesis and the CB1 receptor as a viable treatment strategy. By providing insights into the molecular mechanisms underlying naringin's effects, our findings pave the way for further research into its clinical applications and the development of targeted therapies for ASD.

## 2 Materials and Methods

### 2.1 Animals

C57BL/6J (C57) mice were obtained from the Experimental Animal Centre of the Army Medical University (EACAMU). BTBR T+ Itpr3tf/J (BTBR) male mice were acquired from Jackson Laboratories and bred at EACAMU. The animals were housed in pairs in cages within an environment that maintained a controlled temperature range of 20°C–24°C and a humidity level of 45%–75%, under a 12‐h light/dark cycle with lights turning on at 7 a.m. Both water and food were freely accessible to the mice. All behavioral tests were conducted at 7 weeks of age, corresponding to early adulthood with stable neuroendocrine function and the complete expression of autism‐relevant phenotypes. Only male mice were used in this study for two reasons. Firstly, clinical evidence indicates that ASD has a higher prevalence among males. We chose to use male mice to increase the translational relevance of our study. Secondly, female BTBR mice exhibit less pronounced autism‐like behavioral phenotypes compared to male BTBR mice.

### 2.2 Drug Treatment

Male BTBR and C57 mice, aged 7 weeks, were utilized in this study. The mice were randomly assigned to one of four groups: (1) C57 + Saline (SAL), (2) C57 + naringin (NAR, Aladdin, N107344, Shanghai, China, purity: 95%, 80 mg/kg), (3) BTBR + SAL, and (4) BTBR + NAR (40/80/120 mg/kg). The mice received daily injections of either naringin or an equivalent volume of saline for seven consecutive days. The doses administered ranged from 0 mg/kg to 120 mg/kg intraperitoneally (i.p.) to evaluate the dose‐dependent effects of naringin treatment [19]. We conducted the three‐chambered social, self‐grooming, and marble‐buried tests to assess autistic behaviors. As medium doses of naringin proved to be most effective in alleviating autism‐like behaviors in BTBR mice, we chose to use this dose for subsequent experiments. ACEA (arachidonyl‐2′‐chloroacetamide, HY‐110004, USA, purity: ≥ 96.0%), a potent and highly selective CB1 receptor agonist, an intraperitoneal dose of 0.3 mg/kg [20] (dissolved in a 1% DMSO saline solution) was administered 4 h after the injection of naringin to explore the involvement of CB1 receptor in naringin's therapeutic effects. Specifically, male BTBR mice were given 80 mg/kg of naringin via i.p. injection for 7 consecutive days, starting at 7 weeks of age.

### 2.3 Behavioral Assays

Each behavioral assessment was conducted on the same day. At least 30 min prior to testing, subjects were transferred from their respective colonies to a sound‐attenuated behavioral testing room. Only one assessment was conducted per day until all evaluations were completed. To ensure cleanliness, a 75% ethanol solution was employed to sanitize the apparatus following each trial.

#### 2.3.1 Three‐Chambered Social Approach

A three‐chamber social approach test was used to assess the socialization of mice based on a previously established protocol [21]. Social behavior was assessed in a rectangular three‐chamber apparatus (40 cm × 60 cm × 22 cm), divided into three sections, with retractable doors in the two partitions, allowing the mice free access to each chamber. The test consisted of a 10‐min familiarization phase and a 10‐min social approach phase. During the familiarization phase, a subject mouse was positioned in the central chamber and allowed to explore the arena for 10 min. In the subsequent social approach phase, a novel mouse(S) and object(O) were introduced on each side. The mouse was positioned in the center chamber and given 10 min to explore the three chambers. Total time spent in each chamber, as well as time spent sniffing, were analyzed using Ethovision XT 11.5. The preference index of the chamber or sniffing was calculated, representing the numerical time difference between chambers or sniffing (S minus O) divided by the total time in both chambers or sniffing.

#### 2.3.2 Self‐Grooming Test

Mice were housed in new standard cages without bedding, and their movements were recorded for 20 min using a high‐definition camera. The initial 10 min served as a familiarization phase, followed by a 10‐min testing phase. During the testing phase, the self‐grooming behavior of the mice was analyzed through manual scoring.

#### 2.3.3 Marble Burying Test

The marble burying test was conducted as previously described [22]. Mice were placed in a transparent cage with a 10 cm deep layer of wood chip bedding. Twenty black glass marbles, each with a diameter of 1.5 cm, were arranged in a 4 × 5 grid throughout the cage. The mice were allowed 30 min to bury the marbles. A marble was deemed “buried” if the bedding material covered at least two‐thirds of its surface. The total number of buried marbles was subsequently counted.

#### 2.3.4 Novel Object Recognition Test

Considering the mouse's innate tendency to seek out novelty, it devotes most of its time to exploring a new object upon recognizing the familiar one. As previously outlined, the procedure used standard equipment, specifically a gray plexiglass box measuring 40 cm × 40 cm × 30 cm. The experiment consisted of two distinct phases: an exploratory phase and a testing phase. In the exploratory phase, two identical objects (A), matching in shape, color, size, and texture, were positioned on opposite ends of the diagonal at the base of the apparatus. In the test phase, replace one of the objects with an object (B) that is similar in size but different in shape and color. The mice were then allowed to explore freely for 10 min; their behavior was recorded. After experimenting, the formula DI = (tB/(tA + tB)) × 100% was utilized to calculate the discriminating index (DI). Subsequently, the device was meticulously cleaned using a mixture of 75% alcohol and water.

#### 2.3.5 Y Maze Test

The Y Maze test assesses mice's short‐term spatial working memory capacity by observing spontaneous alternation behavior. The Y maze comprises three compartments, each measuring 30 cm in length, 5 cm in width, and 15 cm in height, arranged at identical angles of 120° to create its structure. Mice are gently placed at the center and are allowed 8 min to explore the maze. Software (Ethovision 11.5 software, Noldus Information Technology) was used to record the number (N) of mouse entries into each arm and correct alternations (N1). Sequential entries into three different arms were considered to be an alternation. Alternation rate (%) = N1/(*N* − 2) × 100%.

#### 2.3.6 Nest Building

Nesting is used to test cognitive abilities in mice. The experimental procedure mainly refers to the previous literature [23]. Mice were placed in clean standard cages, and nearly 2.5 g/5 cm^2^ of square compressed cotton was introduced into the cages. An adequate supply of food and water was given. After 12 h, the quality of the nest was assessed on a 5‐point scale.

#### 2.3.7 Open Field Test

Mice were gently placed in the center of the blank area (40 cm × 40 cm × 30 cm) and allowed to explore freely for 30 min, which was recorded using a high‐definition camera. Finally, the distance traveled and the time spent in the center area were calculated by Ethovision 11.5.

### 2.4 Quantitative Real‐Time PCR (qRT‐PCR)

Hippocampal tissue was removed and immediately placed in liquid nitrogen, then transferred to −80°C. Total RNA was extracted using an RNAeasyTM kit (R0026; Beyotime) following the manufacturer's instructions. Firstly, the lysis buffer was added to the centrifuge tube containing the hippocampal tissue. Then, an equal volume of binding solution was added. Transfer the mixture (including precipitate) to a purification column, centrifuge at 12,000 rpm for 30 s, and discard the liquid in the collection tube. Next, add washing solution I, centrifuge at 12,000 rpm for 30 s, and discard the liquid in the collection tube. Then, add washing solution II and repeat the operation twice as above. Subsequently, the sample underwent centrifugation at 15,000 rpm for 2 min, and the remaining liquid was removed. Finally, place the RNA purification column in the RNA elution tube provided in this kit. Add 30 μL of elution solution, let it stand at room temperature for 2–3 min, and then centrifuge at maximum speed for 30 s. The resulting solution is the purified RNA. Real‐time PCR was carried out after total RNA was extracted and reverse‐transcribed into cDNA using an RT‐PCR kit (Takara, Japan) following the manufacturer's instructions. RT‐PCR analysis was conducted utilizing SYBR Green PCR technology (Takara, Japan). The expression levels of the target genes were normalized to those of Gapdh, and the expression of the target genes was determined using the 2^−ΔΔ*CT*^ method. The sequences of primers are listed in Table 1.

**TABLE 1:** The sequences of primers used in this study.

Gene	Forward primer (5′–3′)	Reverse primer (5′–3′)
Gapdh	CATGGCCTTCCGTGTTCCTA	GCCTGCTTCACCACCTTCTT
Mag	CCTTCAACCTGTCTGTGGAGTTT	CAAACTCCCTCTCCGTCTCATTC
Cdkl5	GCAGACACAAGGAAACACATGAA	TGGCATTTCTTCCAGCAATTCAA
Sema6a	AGACGCATCCACTCATGGAC	ATTCTGATATGGCCCGGCAG
Plxnb3	GTCAGGAACAGGGTCAGATCATT	TCCACCTGAGCTAGACTGTTGTA
Hes5	CATGGCCCCAAGTACCGT	CTCTATGCTGCTGTTGATGCG
Cnr1	AAGTCGATCTTAGACGGCCTT	TCCTAATTTGGATGCCATGTCTC

### 2.5 Western Blot

Hippocampal tissue was removed and immediately placed in liquid nitrogen, then transferred to −80°C for western blot experiments. Hippocampal samples were homogenized using Lysis Buffer (RIPA, Beyotime, China), supplemented with protease inhibitors (PMSF, Thermo, USA) and a phosphatase inhibitor cocktail (Beyotime Institute of Biotechnology, China). Subsequently, the samples were centrifuged at 4°C at 14,000 rpm for 30 min, following which protein concentration was determined using the BCA Kit (Beyotime, China). The protein in the supernatant were separated by 10% SDS‐PAGE for 40 min at 150 V and transferred onto polyvinylidene fluoride (PVDF) membranes (Millipore, USA) with a current of 220 mA for 45 min. The membrane was then blocked for 15 min with a protein‐free rapid‐blocking buffer (Epizyme, PS10). The primary antibodies utilized in this study are listed as follows: the rabbit polyclonal antibodies anti‐CB1 (1:1000, A1447, ABclonal, China), anti‐β‐TUBULIN (1:1000, BM1453, Boster Biological Technology, China), and anti‐DCX (1:1000, Cat.4604S, Cell Signaling Technology). After incubation with the primary antibody, the membrane was incubated in the appropriate secondary antibody solution for 2 h at room temperature. Subsequently, it was exposed using an ECL Kit to reveal the bands corresponding to the target proteins.

### 2.6 Immunofluorescence

Male mice were perfused transcardially with 0.01 M phosphate‐buffered saline (PBS) followed by 4% paraformaldehyde (PFA) for 15–20 min. Whole brains were collected and soaked in 4% PFA for 48 h at 4°C, then dehydrated in a 30% sucrose solution and 4% PFA at 4°C. The 30 μm coronal brain sections were cut using an RWD FS800 cryosectioner and stored in cryoprotectant solution at −20°C. Frozen sections were washed 3 times with PBS and then incubated in a solution containing 0.3% Triton X‐100 and 3% bovine serum albumin (BSA) to block non‐specific binding at room temperature. Next, the frozen sections were incubated overnight with rabbit anti‐DCX (Cell Signaling Technology, 1:500), mouse anti‐SOX2 (SRY‐box transcription factor 2, Abcam, 1:500), and rabbit anti‐GFAP (Glial fibrillary acidic protein, Dako, 1:500). The next day, wash the sections with PBS and apply the secondary antibody. Sections were incubated (2 h, 37°C) with Cy3‐coupled or 488‐coupled secondary antibodies (1:500, Jackson), then counterstained with 4′,6‐diamino‐2‐phenylindole (DAPI, Sigma, 268298). Immunofluorescence images were captured and analyzed utilizing a confocal microscope (Zeiss LMS880).

### 2.7 RNA‐Seq Analyses

Total RNA was extracted from the hippocampal tissue using an RNAeasyTM kit (R0026; Beyotime Biotechnology) following the manufacturer's instructions. Biological replicates consisted of three mice per group in the C57 + SAL, C57 + NAR, BTBR+SAL, and BTBR+NAR groups. The extracted RNA was analyzed for concentration and purity using a NanoDrop2000. A sequencing library was developed and sequenced on the NovaSeq 6000 platform (Illumina) by Shanghai Personal Biotechnology Co. Ltd. We then performed expression analyses using the RSEM software and differentially expressed genes (DEGs) using the DESeq2 software. The parameters of this DEG software are set to fold‐change (FC) > 1.2 and *p*‐value < 0.05. In order to further analyze the DEGs, we used the Gene Ontology (GO) database. Genes were classified according to the Biological Process (BP) they participate in, the Cellular Component (CC) they constitute, and the Molecular Function (MF) they fulfill. GO annotation was performed on the differentially expressed genes. Using the Kyoto Encyclopedia of Genes and Genomes (KEGG) database, genes were classified according to the pathways they participate in or the functions they perform. KEGG annotation was performed on the differentially expressed genes. Finally, we created a clustered heat map of the genes of interest to represent the differences in gene expression between groups. All the raw sequences were deposited in the NCBI Sequence Read Archive (SRA) under the accession number PRJNA1228256.

### 2.8 Cell Counting of DCX ^+^, SOX2 ^+^, and SOX2 ^+^/GFAP ^+^ Positive Cells

Radial glial cells (RGCs) were subjected to immunofluorescent double staining using SOX2 and GFAP. The processes labeled with GFAP typically extend into the molecular layer. The quantities of SOX2^+^, SOX2^+^/GFAP^+^, and DCX^+^ cells within the SGZ were quantified across five matched sections from each mouse, and the average count for each section was calculated for all subjects. The analysis included five mice from each group.

### 2.9 Statistical Analysis

Paired *t*‐tests were used in the three‐chamber socialization test to compare the time mice spent in the different chambers. Two‐way ANOVA with the least significant difference (LSD) post hoc test was used to compare the differences among the four groups. In contrast, comparisons between the other groups were made using a one‐way ANOVA with LSD's post hoc test. For the open‐field tests, repeated‐measures ANOVA was employed to assess the following factors: time (within‐group factor), mouse strain (between‐group factor), and drug treatment (between‐group factor). All data were analyzed using SPSS 23.0 and presented as the mean ± standard error of the mean (SEM). *ɑ* = 0.05; *p < 0.05, **p < 0.01, ***p < 0.001.

## 3 Results

### 3.1 Naringin Treatment Rescued Cognitive Impairment but Did Not Influence Locomotor Ability or Anxiety Behaviors in BTBR Mice

**FIGURE 1:**
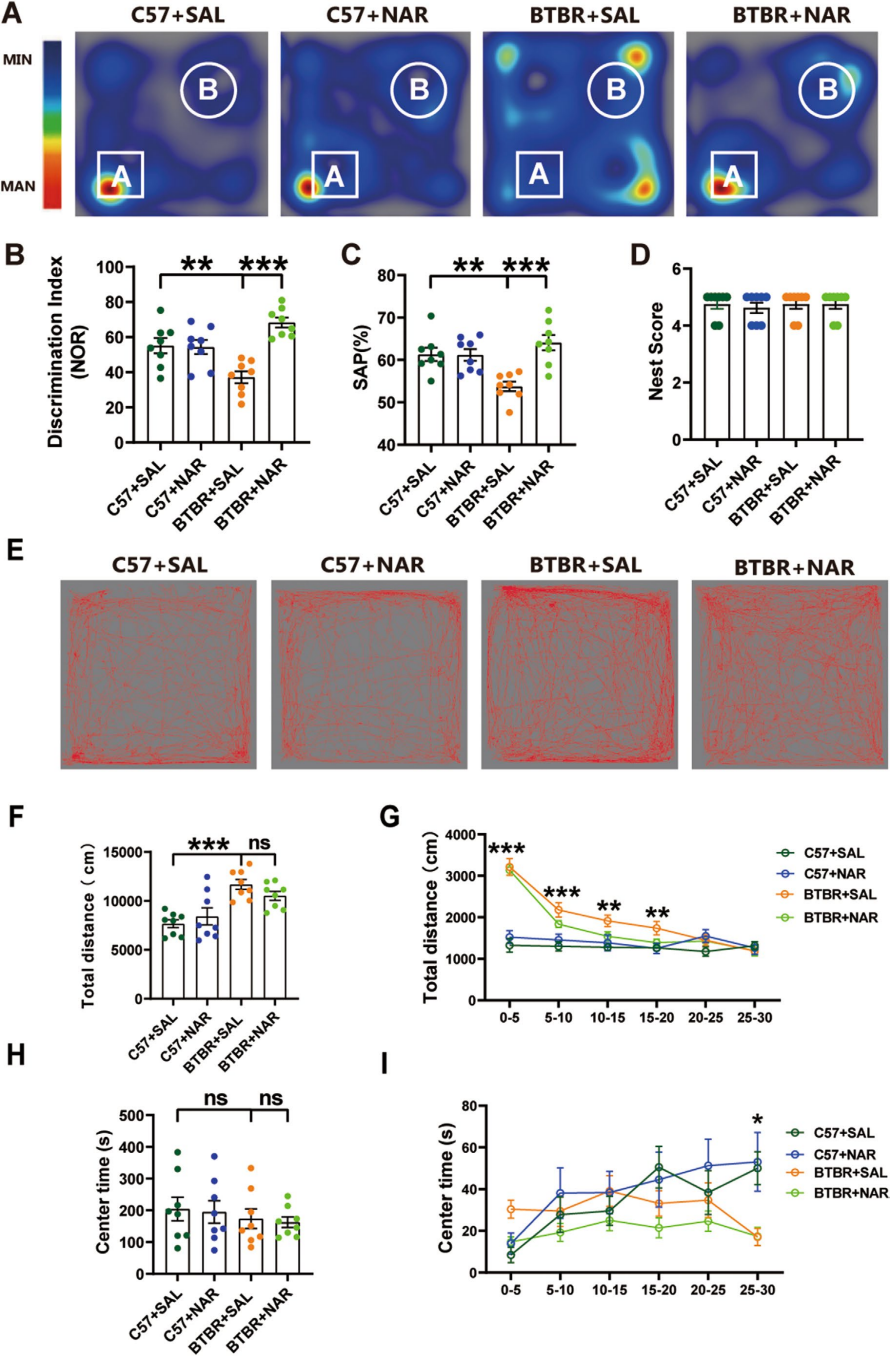
Naringin treatment rescued cognitive impairment but did not influence locomotor ability or anxiety behaviors in BTBR mice. (A) Representative heat maps showing the total time and position of C57 and BTBR mice in a novel object recognition test. “A” and “B” represent novel and familiar objects. (B) The recognition index of BTBR mice was lower than that of C57 mice, but this difference was reversed by naringin treatment. (C) The SAP of the BTBR was lower than that of C57 mice in the Y maze test, which was reversed by naringin treatment. (D) Nest score in nesting test of four groups of mice. (E) Representative trajectory diagrams illustrating the movement of four groups of mice within a 30‐min duration during the open‐field test. (F) Total distance traveled in 30 min. (G) The total distance is over 30 min in 5‐min bins. (H) The center time traveled in 30 min. (I) The center time is over 30 min, divided into 5‐min increments. Data are expressed as mean ± SEM. *N* = 8. *p < 0.05, **p < 0.01, ***p < 0.001.

Following the identification of 80 mg/kg as the most effective dose for behavioral improvements in autistic‐like phenotypes (Figure S1), BTBR mice in this cognitive assessment cohort received the exact optimal dosage of naringin (80 mg/kg). This dose selection allowed us to specifically evaluate whether the behavioral improvements observed at this concentration correlated with cognitive enhancement. Memory functions as a critical indicator of cognitive ability, and the effects of naringin treatment were evaluated by using Novel Object Recognition (NOR) test and the Y maze test, which rely on the hippocampus to varying degrees. In the hippocampus‐dependent NOR test, a cognitive deficit was observed in BTBR mice compared to the control group (Figure 1A). Subsequent analysis indicated that the BTBR mice exhibited a significantly lower discrimination index than the C57 mice (*p* < 0.01, Figure 1B). However, treatment with naringin resulted in a marked enhancement of the discrimination index in the BTBR mice, suggesting that naringin may effectively improve impaired short‐term spatial memory in these mice (*p* < 0.001, Figure 1B). In the Y maze spontaneous alternation test, a distinctive defect was noted, characterized by a diminished rate of spontaneous alternation accuracy in BTBR mice, in contrast to the C57 counterparts (*p* < 0.01, Figure 1C). This deficit was ameliorated following naringin treatment (*p* < 0.001, Figure 1C). Furthermore, in the nest‐building test, a reliable measure of hippocampal‐mediated cognitive function, no significant differences were observed between the C57 and BTBR mice (Figure 1D).

Given that locomotor and exploratory behaviors could confound the sociability and repetitive behaviors assessment, we conducted the open‐field test (Figure 1E–G). The distance traveled by the mice decreased in each 5‐min segment over time as the mice increasingly acclimated to the open‐field area (Figure 1G). In the open‐field area, the BTBR mice traversed a longer distance over the 30 min compared to the C57 mice (*p* < 0.001, Figure 1F) as well as during the initial 10 min (*p* < 0.001, Figure 1G) and 10–20 min (*p* < 0.01, Figure 1G). To assess anxiety behaviors, we measured the time that the mice spent in the central area of the open field after they had adapted to the environment. No significant difference was observed between the C57 and BTBR mice (Figure 1H,I). These results suggest that alterations in motor function or anxiety did not confound the behavioral improvements observed in BTBR mice treated with naringin.

### 3.2 Naringin Modified the Transcriptome of the Hippocampus in Four Distinct Groups of Mice, and the Regulation of Neurogenesis Is an Important Mechanism

**FIGURE 2:**
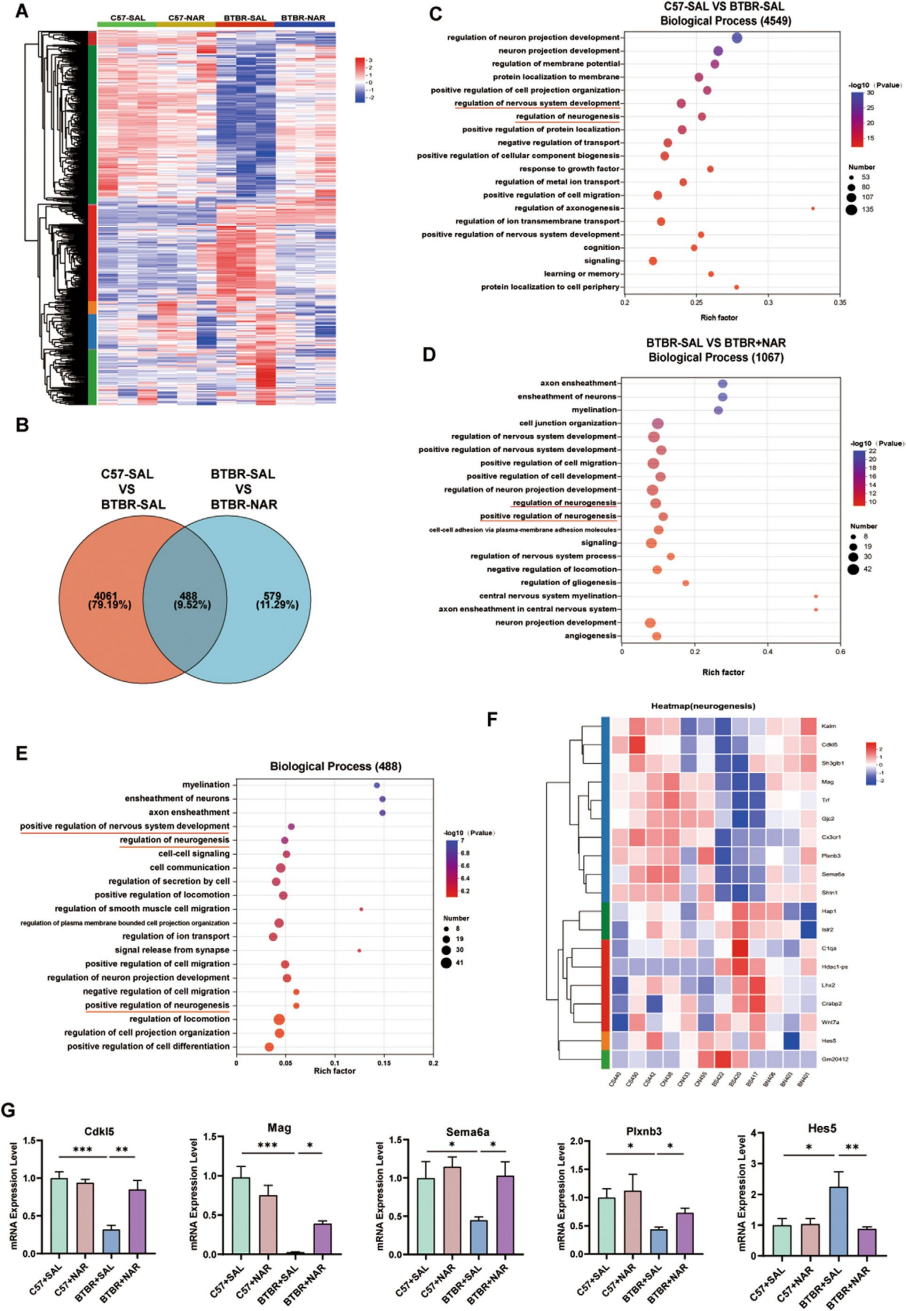
Naringin modified the transcriptome of the hippocampus in four distinct groups of mice. (A) The heat map depicts the hierarchical clustering of the four groups. (B) Venn diagram illustrating the treatment of co‐regulated DEGs by NAR. (C) The top 20 GO pathways from C57‐SAL VS BTBR‐SAL in BP enrichment. (D) The top 20 GO pathways from BTBR‐SAL VS BTBR‐NAR in BP enrichment. (E) The top 20 GO pathways from the intersection of the above two gene sets as determined by BP enrichment. (F) The heat map depicts genetic changes related to neurogenesis in the four groups. (G) Verification of high variation genes in RNA‐seq by RT‐PCR. *N* = 3–4. *p < 0.05, **p < 0.01, ***p < 0.001.

A high‐throughput RNA sequencing analysis was performed to investigate the underlying mechanisms of naringin therapy and identify genes exhibiting altered expression profiles in the hippocampus. The hierarchical clustering heat map highlights the differences in gene expression across various subgroups (Figure 2A). The Venn diagram indicates 4549 differentially expressed genes (DEGs) regulated by BTBR when comparing C57 and BTBR‐SAL mice. In addition, 1067 DEGs are regulated explicitly by naringin between the BTBR‐SAL and BTBR‐NAR (naringin) mice. In comparison, 488 DEGs emerge as co‐regulated by both BTBR and naringin, highlighting a fascinating overlap in their regulatory effects (Figure 2B). Gene Ontology (GO) enrichment analysis conducted on the 4549 DEGs identified significant enrichment in a variety of critical biological processes, including the regulation of neuron projection development, neuron projection development, positive regulation of nervous system development, and the regulation of neurogenesis (Figure 2C). The functional analysis of the 1067 DEGs identified between the BTBR‐SAL and the BTBR‐NAR groups primarily highlighted enriched GO terms related to the regulation of nervous system development, positive regulation of nervous system development, regulation of neurogenesis, and positive regulation of neurogenesis (Figure 2D). Additionally, the functional analysis of the 488 DEGs that were co‐regulated by BTBR and naringin revealed that the GO terms predominantly encompassed the regulation of nervous system development, positive regulation of nervous system development, and regulation of neurogenesis (Figure 2E). The term “regulation of neurogenesis” was notably identified across three distinct gene sets, suggesting that this pathway may play a critical role in the therapeutic effects of naringin. Consequently, we constructed a heatmap illustrating the genes associated with the regulation of neurogenesis (Figure 2F) and subsequently selected several genes for validation through RNA sequencing (Figure 2G). The results obtained from the qRT‐PCR analysis corroborated the trends observed in the heatmap, demonstrating consistency in the findings. These results indicate that hippocampus neurogenesis contributes to the therapeutic effects of naringin in the BTBR mouse model of autism.

### 3.3 Naringin Treatment Enhanced DG Neurogenesis in BTBR Mice

**FIGURE 3:**
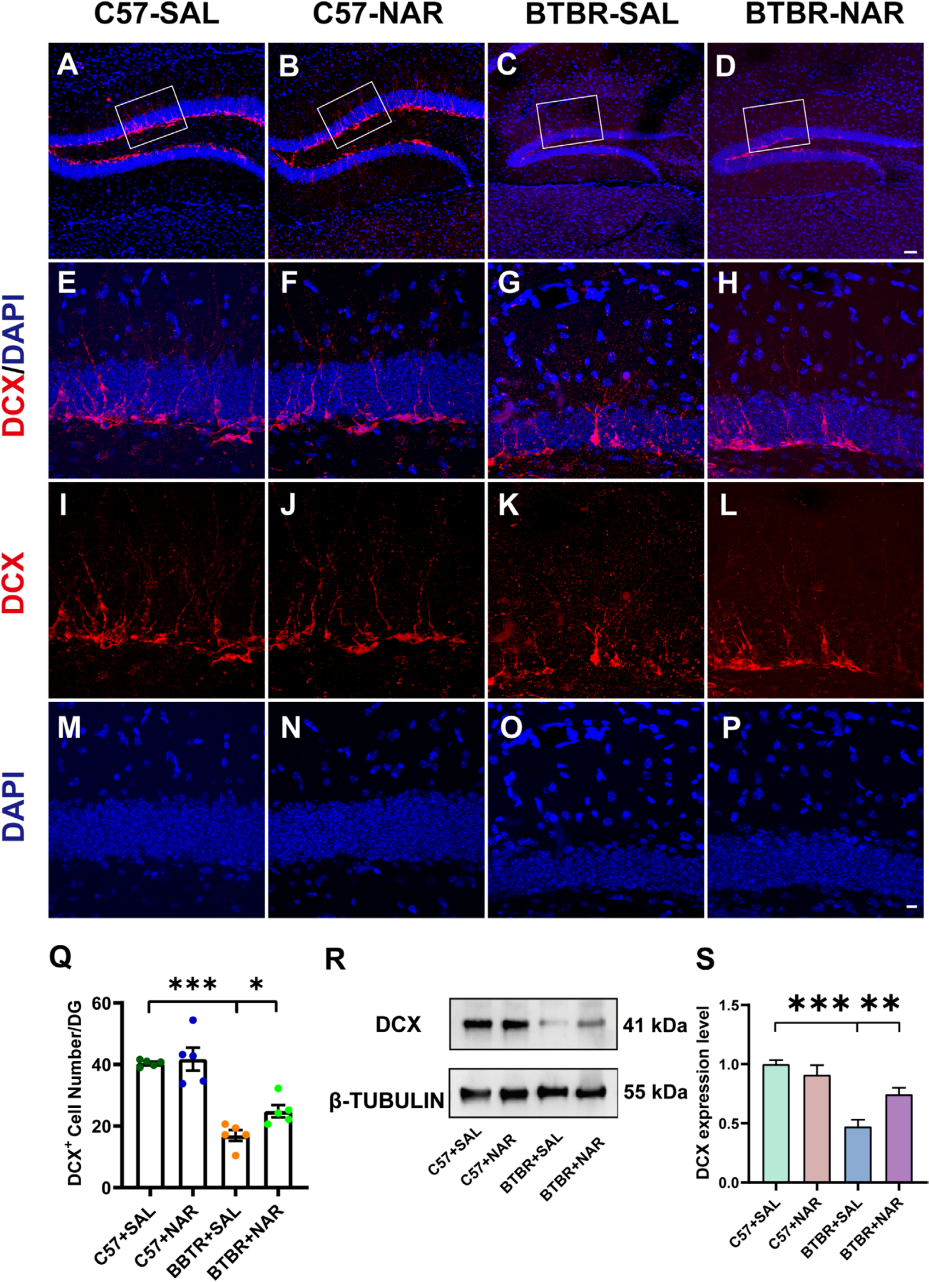
Naringin treatment enhanced DG neurogenesis in BTBR mice. (A–D) Representative images of DCX‐positive cells in the DG of four groups of mice. (E–P) High‐magnification images showing immunolabeled DCX (in red), DAPI (in blue), and colocalization of DCX^+^/DAPI^+^ cells. (Q) Quantifying the number of DCX^+^ cells in the DG. *N* = 5. (R) Representative Western blot analysis of DCX expression in the hippocampus of mice within each group. (S) Quantification of DCX protein level in the hippocampus. *N* = 4. The scale bar = 50 μm is shown in (D) and applied to (A–D). The scale bar = 10 μm, is shown in (P) and applied to (E–P). Data are presented as mean ± SEM. *p < 0.05, **p < 0.01, ***p < 0.001.

Our previous studies have confirmed that BTBR mice exhibit defects in hippocampal neurogenesis [24]. Additionally, the RNA sequencing results from this study suggest that neurogenesis plays a significant role in mediating these observed abnormalities. To evaluate the impact of naringin on hippocampal neurogenesis, we selected a specific marker: DCX, which labels immature neurons in the DG granule cell layer, highlighting the dynamics of neuronal development. DCX is a microtubule‐associated protein essential for the migration and differentiation of neurons, primarily expressed in NPCs within the developing and mature central nervous system. The migration of NPCs is closely associated with DCX expression, and the protein is also present in differentiating neurons. This highlights its significance for neuronal plasticity, axon outgrowth, and synthesis. Our study found a notable reduction in the number of DCX^+^ cells in BTBR mice (Figure 3A–P; *p* < 0.001, Figure 3Q). This finding is consistent with earlier research conducted in our laboratory, reinforcing the idea of altered neurogenesis in these models. Remarkably, following treatment with naringin, there was a significant increase in the number of DCX^+^ cells within the BTBR mice (*p* < 0.05, Figure 3Q), suggesting a potential therapeutic effect of naringin on neurogenesis. We further assessed DCX protein expression using western blotting across the four experimental groups (Figure 3R). Among the four experimental groups, DCX protein expression levels in the BTBR‐SAL group were significantly decreased (*p* < 0.001, Figure 3S). However, this trend was reversed following naringin treatment (*p* < 0.01, Figure 3S).

### 3.4 Naringin Treatment Rescues the Pool of NPC in the DG of BTBR Mice

**FIGURE 4:**
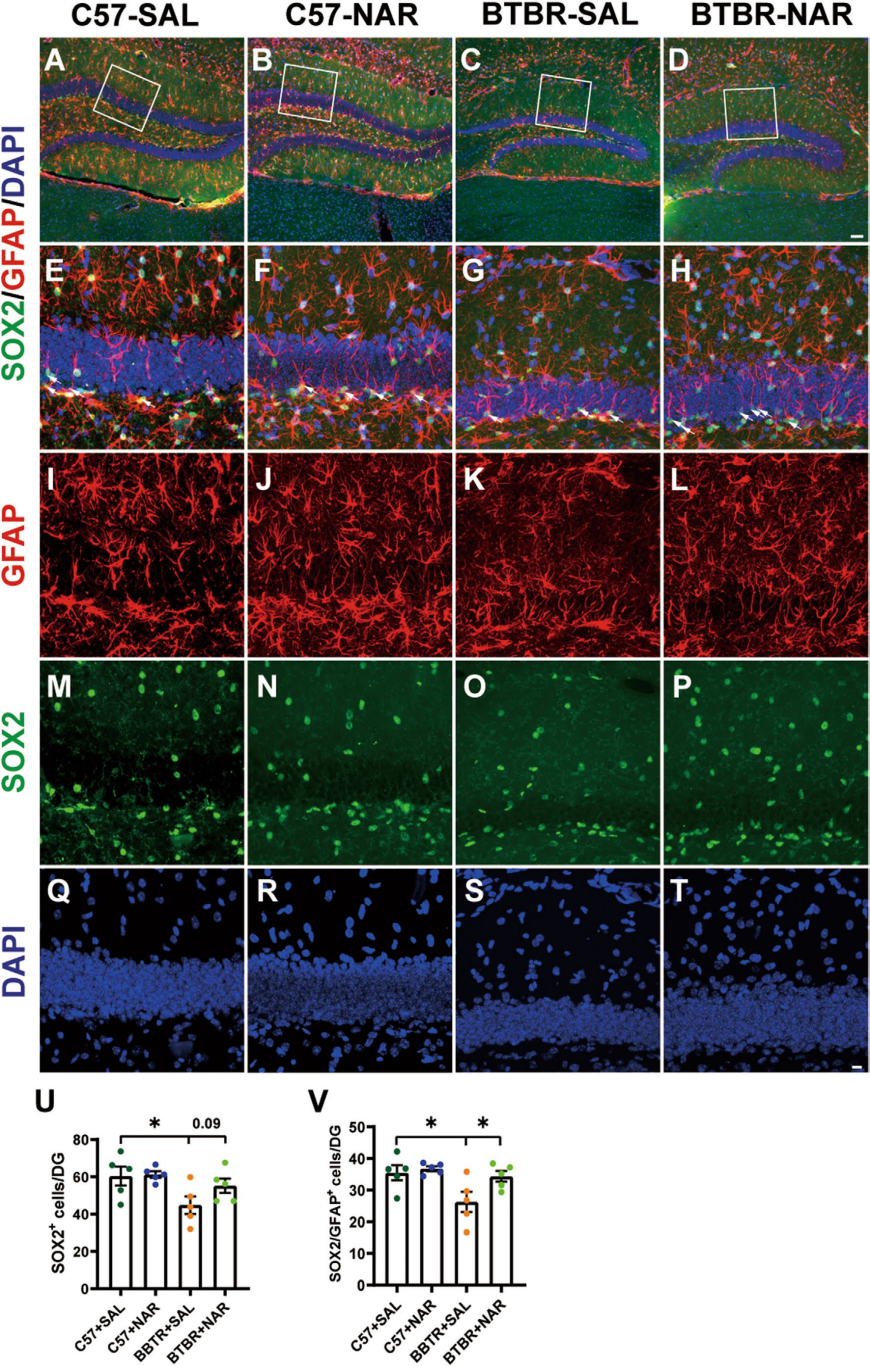
Naringin treatment rescues the pool of NPC in the DG of BTBR mice. (A–D) Representative images of SOX2^+^/GFAP^+^ double‐positive NPCs in the DG of four groups of mice. (E–T) High‐magnification image of the designated area. (U) Quantification of the number of SOX2^+^ cells in the DG. (V) Quantification of SOX2^+^/GFAP^+^ cells in the DG. The scale bar = 50 μm is shown in Figure D and applied to (A–D). The scale bar = 10 μm is shown in Figure T and applied to (E–T). Data are presented as mean ± SEM. *N* = 5. *p < 0.05.

Emerging evidence indicates that various methodologies may significantly enhance neurogenesis in the hippocampus and mitigate behavioral symptoms associated with ASD in animal models [25]. The transcription factor SOX2 plays a pivotal role in sustaining the pluripotency of NPCs and promoting their proliferation, primarily within the SGZ of the DG [26]. Our analysis revealed notable alterations in the population of SOX2^+^ cells across four distinct groups of mice (Figure 4A–U). Notably, treatment with naringin was associated with a promising upward trend in the number of SOX2^+^ cells within the hippocampus of BTBR mice, approaching statistical significance (*p* = 0.09, Figure 4A–U). NPCs are mainly derived from RGCs in the DG‐SGZ of the adult hippocampus, typically identified through double staining with GFAP and SOX2. RGCs are distinguished by their striking vertical radial processes that extend across the granule cell layer (GCL). Notably, the quantity of SOX2^+^/GFAP^+^ RGCs plays a crucial role in determining the size of the NPCs pool. The findings showed that the NPCs pool in the hippocampal DG of BTBR mice was significantly diminished compared to that of C57 mice, with statistical significance (*p* < 0.05, see Figure 4V). However, treatment with naringin has markedly enhanced the NPCs population in BTBR mice (*p* < 0.05, see Figure 4V). These results imply that naringin treatment has the potential to mitigate the diminished NPC pool observed in the hippocampal DG of BTBR mice, opening avenues for further research into therapeutic strategies for neurogenesis.

### 3.5 Naringin Treatment Inhibits eCBs in the Hippocampus of BTBR Mice

**FIGURE 5:**
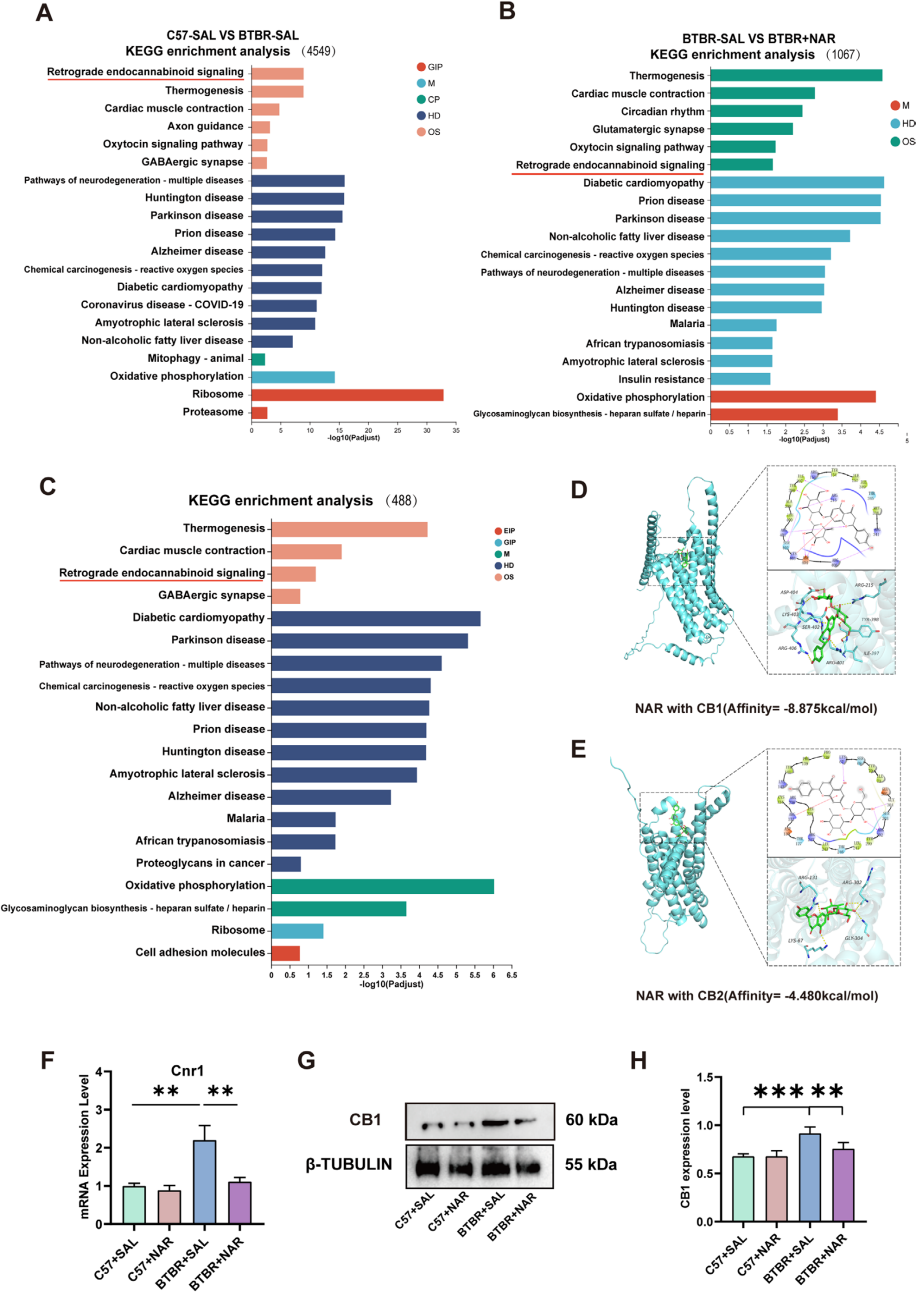
Naringin treatment inhibits eCBs in the hippocampus of BTBR mice. (A) The top 20 GO pathways from C57‐SAL VS BTBR‐SAL in KEGG analysis. (B) The top 20 GO pathways from BTBR‐SAL VS BTBR‐NAR in KEGG analysis. (C) The top 20 GO pathways from the intersection of the above two gene sets in KEGG analysis. (D) Verification of naringin and CB1 receptor by molecular docking analysis. (E) Verification of naringin and CB2 receptor by molecular docking analysis. (F) The expression level of Cnr1 gene in RNA seq by RT‐PCR. (G) The expression level of CB1 receptor protein in western blot. (H) Quantification of the CB1 receptor protein level in the hippocampus. Data are presented as mean ± SEM. *N* = 4. *p < 0.05, **p < 0.01, ***p < 0.001.

We performed KEGG enrichment analysis across the three groups to investigate the factors influencing neural development. The results from all three enrichment analyses consistently identified the retrograde endocannabinoid signaling (eCBs) as a significant focus, prominently featured in all analyses (Figure 5A–C). We hypothesized that naringin could influence neurogenesis in the DG region of the hippocampus in BTBR mice through its effect on the eCBs. The eCBs interact with two primary receptors: CB1 and CB2. Notably, the CB1 receptor is recognized as the G protein‐coupled receptor (GPCR) with the highest expression in the central nervous system. To further investigate, we conducted a molecular docking analysis to examine the interaction between naringin and CB1 or CB2 receptors. The computational docking simulations revealed that naringin exhibited a significantly stronger binding affinity for the CB1 receptor (Δ*G* = −8.875 kcal/mol) compared to the CB2 receptor (Δ*G* = −4.480 kcal/mol) (Figure 5D,E). These results suggest that naringin interacts more favorably with the CB1 receptor. We assessed the expression levels of the CB1 receptor using RT‐PCR and western blot techniques (Figure 5F–H). Our findings revealed a substantial increase in both gene (*p* < 0.01, Figure 5F) and protein (*p* < 0.001, Figure 5G,H) expression levels of the CB1 receptor in BTBR mice compared to the C57 strain. However, following the administration of naringin, these elevated levels were notably reduced, aligning closely with those observed in C57 mice at the mRNA (*p* < 0.01, Figure 5F) and protein levels (*p* < 0.01, Figure 5G,H). These results suggest that naringin may enhance hippocampal DG neurogenesis in BTBR mice by inhibiting the expression of CB1 receptors in the retrograde cannabinoid receptor signaling pathway.

### 3.6 ACEA Blocked the Improved Effect of Naringin in BTBR Mice

**FIGURE 6:**
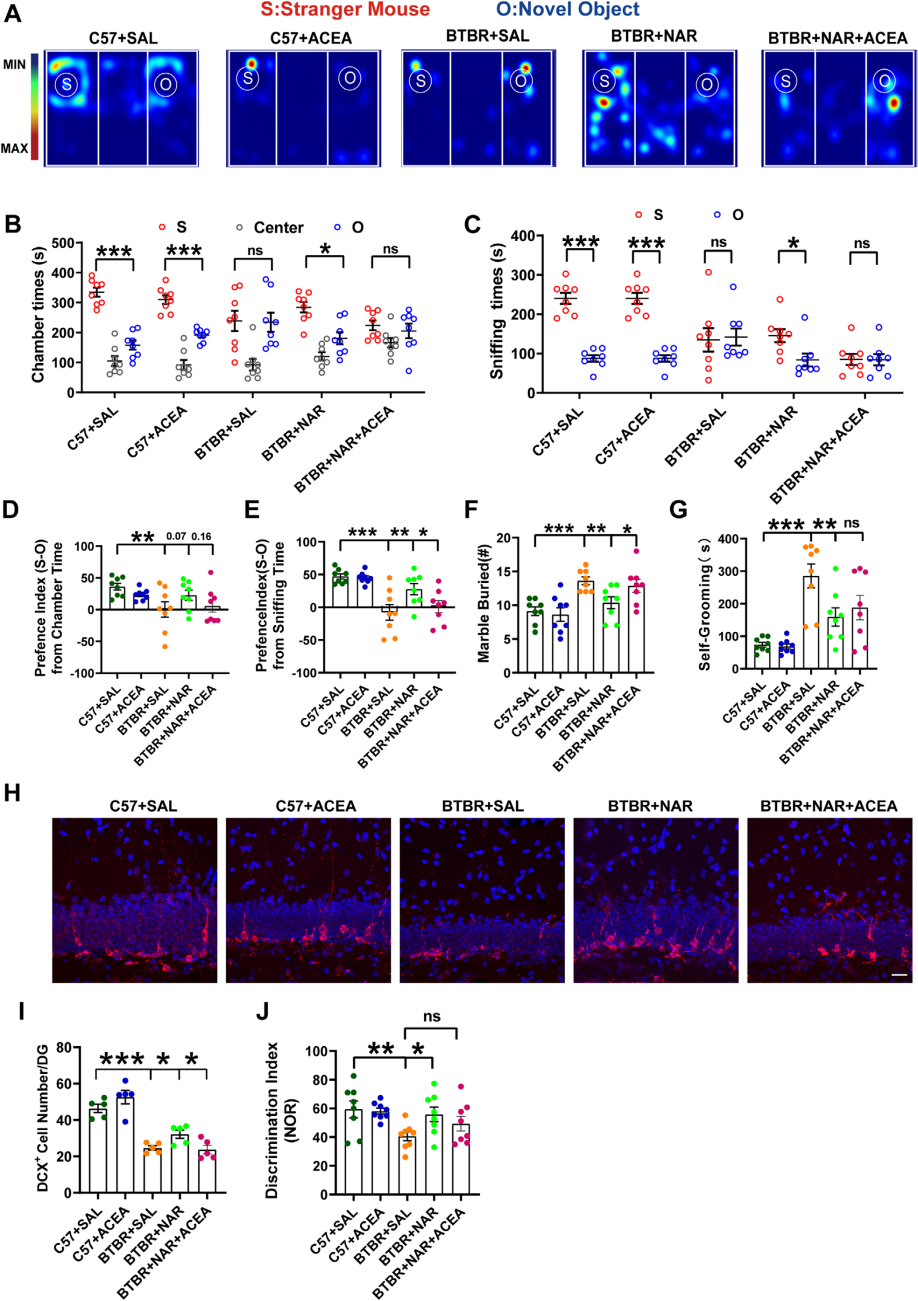
Excitement of CB1 reversed the improvement effects of naringin in BTBR mice. (A) Representative heat maps of the social recognition test in the three‐chamber social test. Warmer colors (red) indicate that the experimental mice spent much time exploring. “S” and “O” represent the novel mouse and object, respectively. (B) Time spent in the three chambers during the 10‐min test. (C) Time spent sniffing the novel mouse (S) or object (O). (D) The preference index (S‐O) from chamber time. (E) The preference index (S‐O) from sniffing time. (F) Statistic of the number of buried marbles. (G) Time spent in self‐grooming. (H) High‐magnification images showing immunolabeled colocalization of DCX^+^(red)/DAPI^+^(blue) cells. (I) Quantifying the number of DCX^+^ cells in the DG (*n* = 5 mice per group). (J) The recognition index of BTBR and C57 mice. The scale bar = 20 μm. Data are presented as mean ± SEM. *N* = 8. *p < 0.05, **p < 0.01, ***p < 0.001.

Considering the significant reduction in CB1 receptor expression observed with naringin treatment, we hypothesized that the CB1 receptor might play a crucial role in mediating the protective effects of naringin against neurogenetic defects in the BTBR mouse model. To explore the underlying mechanisms of naringin's neuroprotective action, we employed ACEA, a highly selective and potent CB1 receptor agonist [27], to further investigate its effects. Behavioral assessments conducted on adult experimental mice demonstrated that naringin treatment was effective across various behavioral tests, including the three‐chambered social approach (Figure 6A–E), marble‐burying (*p* < 0.01, Figure 6F), self‐grooming (*p* < 0.01, Figure 6G), and the NOR test (*p* < 0.05, Figure 6J). In addition, the number of DCX^+^ cells in BTBR mice significantly increased following naringin treatment (Figure 6H,I). However, it is critical to note that these positive effects were significantly inhibited by the administration of ACEA, except for the self‐grooming test (Figure 6A–J), suggesting that the activation of the CB1 receptor may counteract the neuroprotective properties of naringin. This finding highlights the intricate relationship between cannabinoid signaling and neuroprotection, underscoring the need for further investigation into this connection. Furthermore, administering ACEA to adult C57 mice yielded no discernible phenotypic abnormalities, suggesting a 0.3 mg/kg ACEA dosage is comparatively safe (see Figure 6 for visual reference). These compelling findings imply that naringin plays a crucial role in mitigating deficits in hippocampal neurogenesis through the mechanisms of retrograde endocannabinoid signaling. This pathway ultimately contributes to restoring social behavior, alleviating stereotypic actions, and improving cognitive impairments observed in the BTBR mouse strain.

## 4 Discussion

The current study provides compelling evidence that administering naringin could significantly improve ASD‐related behavioral symptoms in BTBR mice. Notably, naringin treatment led to a remarkable enhancement in sociability among these mice, and displayed a pronounced preference for interacting with novel mice, which suggests a shift toward more social behaviors. Furthermore, naringin administration effectively mitigated repetitive and stereotypic behaviors in BTBR mice, as evidenced by a reduction in buried marbles and a decrease in self‐grooming time. Notably, the beneficial behavioral changes observed following naringin treatment were achieved without adversely affecting the mice's locomotor skills or levels of anxiety. This highlights the potential of naringin as a therapeutic agent. Additionally, it is worth noting that no adverse side effects were reported in the C57 mice that received naringin supplementation, further supporting the safety and efficacy of naringin in this experimental context.

Our comprehensive analysis of RNA sequencing data obtained from the hippocampal region provides compelling evidence that naringin treatment significantly impacts the regulation of neurogenesis and the development of the nervous system. Hippocampal neurogenesis, particularly in the DG, is critical for cognitive [28] and behavioral functions [29], and any disruption in this process has been implicated in the pathophysiology of ASD [30]. Following the administration of naringin treatment, we observed a remarkable restoration of impaired hippocampal neurogenesis, which could be fundamental to the observed behavioral improvements. This enhancement was substantiated by a notable increase in the population of DCX‐positive neurons in the DG of the hippocampus, indicating a significant boost in neuronal development and maturation. Moreover, our findings indicated that naringin treatment also profoundly expanded the populations of NPCs in the DG of BTBR mice. This was particularly evident through a marked increase in the number of cells co‐expressing SOX2 and GFAP, which are key markers indicative of NPCs. These compelling results suggest that enhancing hippocampal neurogenesis resulting from naringin treatment may be instrumental in improving autism‐like behaviors, offering a promising avenue for therapeutic interventions.

RNA sequencing analysis further elucidated the molecular mechanisms underlying naringin's effects. Notably, we identified the involvement of the eCBs in mediating the therapeutic actions of naringin. Recent research has suggested that eCBs, which serve as central retrograde messengers in the brain, can directly or indirectly influence neurogenesis in the adult hippocampus [31]. These eCBs primarily serve as endogenous agonists for the metabotropic cannabinoid receptors CB1 and CB2 [32], with the CB1 receptor being the most abundantly expressed in the central nervous system [33], particularly in the adult hippocampus. Studies involving mice lacking the CB1 receptor have revealed notable alterations in NPCs dynamics [34]. Specifically, these studies demonstrate a marked reduction in the proliferation of NPCs, alongside a significant decline in the differentiation and survival rates of their cellular offspring [35]. Contrary to these findings, several studies indicate that the pharmacological blockade of the CB1 receptor can result in an unexpected increase in cell proliferation within the SGZ [36]. This paradox highlights the complex role that the CB1 receptor plays in neural development and function.

Our research revealed a significant elevation in the expression of CB1 receptor mRNA and protein levels within the hippocampus of BTBR mice, indicating a marked alteration in cannabinoid receptor dynamics. Notably, this increase could be effectively suppressed through treatment with naringin, suggesting a potential therapeutic avenue. Furthermore, the findings derived from CB1 receptor intervention using ACEA underscored the critical role of the CB1 receptor in mediating the effects of naringin, which appears to play a pivotal role in alleviating autistic behaviors observed in BTBR mice. Following treatment with MK‐801, zebrafish displayed notable deficits in social communication, accompanied by a significant upregulation of the CB1 receptor within the telencephalon [37]. In addition, inhibiting the CB1 receptor in male Fmr1 knockout mice (Fmr1(−/y)), employing pharmacological and genetic approaches, led to a remarkable restoration of cognitive function [38]. This intervention also mitigated the overactivation of mTOR signaling pathways and brought about notable changes in spine morphology, suggesting potential avenues for therapeutic interventions. These findings align with growing evidence linking eCBs to behaviors associated with ASD, further emphasizing the potential of targeting the CB1 receptor as a viable therapeutic strategy.

The behavioral improvements observed in naringin‐treated BTBR mice, coupled with the restoration of hippocampal neurogenesis, provide compelling evidence for the therapeutic potential of naringin in ASD. However, it is important to acknowledge the limitations of this study. First, while BTBR mice serve as a valuable model for investigating the complexities of ASD, they do not completely encapsulate the intricate nature of human ASD. To reinforce the validity of these findings, future research should aim to replicate these results in alternative ASD models or clinical populations. Second, despite identifying the CB1 receptor as a pivotal component in mediating naringin's effects, the precise molecular pathways activated downstream of the CB1 receptor remain largely unexplored. Additional mechanistic studies are necessary to explore these pathways and to uncover further molecular targets that could enhance our understanding of naringin's role in ASD treatment.

## 5 Conclusion

In conclusion, our study demonstrates that naringin treatment can alleviate core symptoms associated with ASD. In addition, the application of ACEA, a CB1 receptor agonist, counteracted the effects of naringin treatment. This study highlights the therapeutic potential of naringin and offers new insights into CB1 receptor‐regulated neurogenesis in the hippocampus related to ASD. Future research should further explore the mechanisms underlying the interaction between naringin and the CB1 receptor in regulating neurogenesis and investigate the potential of naringin as a therapeutic agent in clinical settings.

## Author Contributions

**Jing Luo:** methodology. **Yulong Liu:** conceptualization, data curation, formal analysis, methodology, writing – original draft. **Xinggao Zhang:** data curation. **Lian Wang:** data curation. **Tianyao Liu:** methodology. **Meiling Xia:** conceptualization, methodology, investigation, visualization, writing – review and editing. **Jiayin Liu:** data curation. **Mei Chen:** data curation. **Xiaotang Fan:** conceptualization, writing – review and editing, supervision, funding acquisition. **Hong Gong:** formal analysis. **Meifeng Gong:** data curation. **Jinghui Zhao:** formal analysis. **Yi Luo:** conceptualization, methodology, investigation, visualization.

## Ethics Statement

All experimental procedures were approved by the Laboratory Animal Welfare and Ethics Committee of the Army Medical University (AMUWEC20240073). Every effort was made to minimize the number of animals used and to alleviate any potential suffering they may have experienced.

## Conflicts of Interest

The authors declare no conflicts of interest.

## Supporting information


**Figure S1:** The administration of 80 mg/kg naringin (NAR) significantly affected BTBR mice. (A) Representative heat maps showing the total time and position of C57 and BTBR mice in a 10‐min three‐chamber social test. (B) Time spent in the three chambers during the 10‐min test. (C) Time spent sniffing the novel mouse (S) or object (O). (D) The preference index (S‐O) from chamber time in a social novelty recognition test. (E) The preference index (S‐O) from sniffing time in a social novelty recognition test. (F) The number of buried marbles. (G) The time of self‐grooming. Data are expressed as mean ± SEM. *N* = 8. *p < 0.05, **p < 0.01, ***p < 0.001.

## Data Availability

The data that support the findings of this study are available from the corresponding author upon reasonable request.
