# Supplementary figures and images for: Naringin Alleviates Autistic‐Like Behaviors in BTBR Mice Through Cannabinoid Receptor Type 1‐Mediated Restoration of Hippocampal Neurogenesis
Source: CNS Neurosci Ther. 2025 Nov 28;31(12):e70654. doi: 10.1111/cns.70654 (PMC12662764; doi:10.1111/cns.70654)

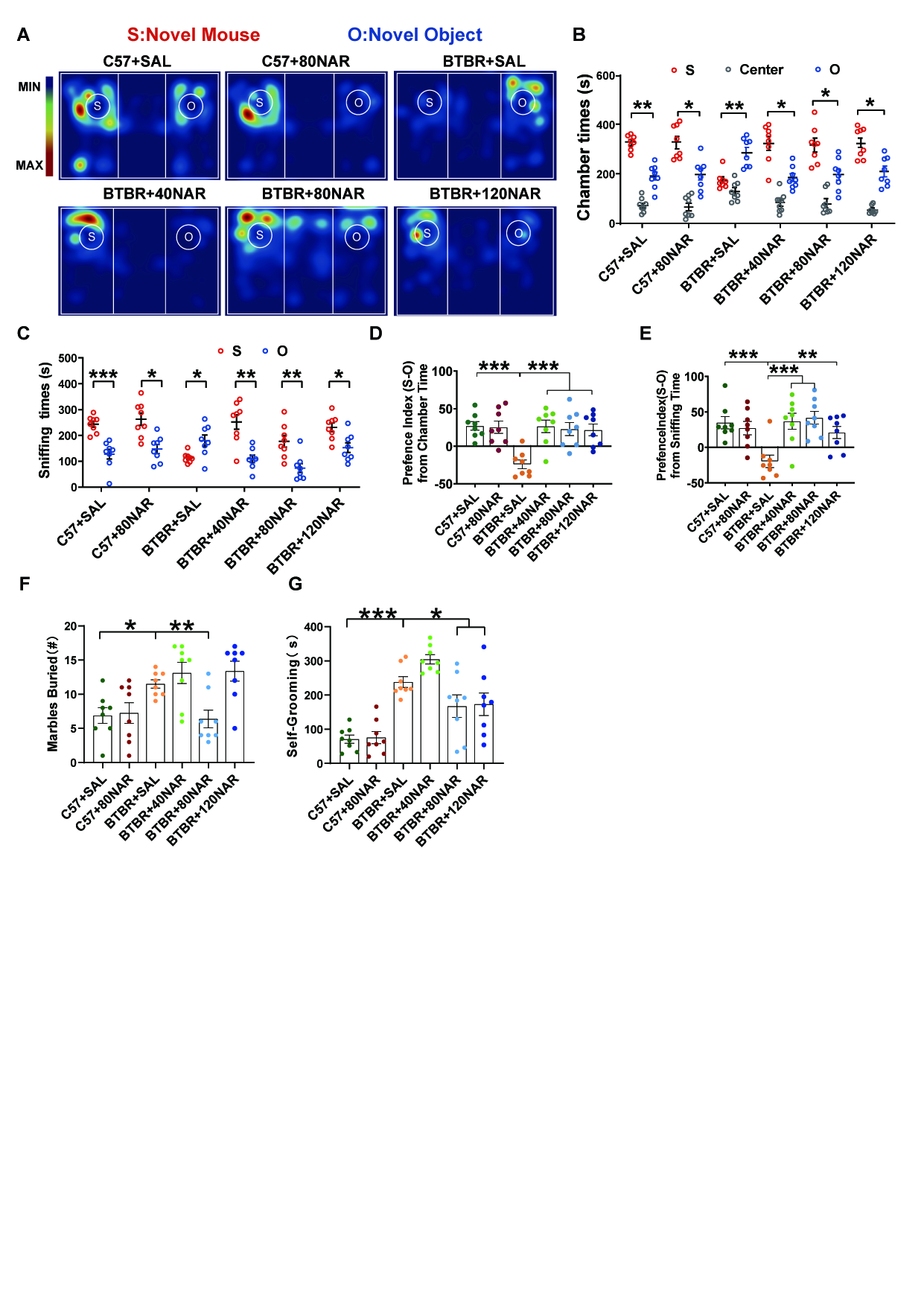

Supplement: Supplementary file 1 — Figure S1: The administration of 80 mg/kg naringin (NAR) significantly affected BTBR mice. (A) Representative heat maps showing the total time and position of C57 and BTBR mice in a 10‐min three‐chamber social test. (B) Time spent in the three chambers during the 10‐min test. (C) Time spent sniffing the novel mouse (S) or object (O). (D) The preference index (S‐O) from chamber time in a social novelty recognition test. (E) The preference index (S‐O) from sniffing time in a social novelty recognition test. (F) The number of buried marbles. (G) The time of self‐grooming. Data are expressed as mean ± SEM. N = 8. *p < 0.05, **p < 0.01, ***p < 0.001. [file CNS-31-e70654-s001.tif]
